# Emergence of liquid following laser melting of gold thin films

**DOI:** 10.1107/S2052252523009363

**Published:** 2023-10-31

**Authors:** Ian K. Robinson, Jack P. Griffiths, Robert Koch, Tadesse A. Assefa, Ana F. Suzana, Yue Cao, Sungwon Kim, Dongjin Kim, Heemin Lee, Sunam Kim, Jae Hyuk Lee, Sang-Youn Park, Intae Eom, JaeHyun Park, Daewoong Nam, Sangsoo Kim, Sae Hwan Chun, Hyojung Hyun, Kyung-Sook Kim, Ming Lu, Changyong Song, Hyunjung Kim, Simon J. L. Billinge, Emil S. Bozin

**Affiliations:** a Condensed Matter Physics and Materials Science Division, Brookhaven National Laboratory, Upton, NY 11793, USA; b London Centre for Nanotechnology, University College London, London WC1E 6BT, United Kingdom; c Materials Science Division, Argonne National Laboratory, Lemont, IL 60439, USA; d Department of Physics, Sogang University, Seoul 04107, Republic of Korea; e Department of Physics and POSTECH Photon Science Center, Pohang University of Science and Technology, Pohang 37673, Republic of Korea; f Pohang Accelerator Laboratory, Pohang, Gyeongbuk 37673, Republic of Korea; g Center for Functional Nanomaterials, Brookhaven National Laboratory, Upton, NY 11793, USA; h Department of Applied Physics and Applied Mathematics, Columbia University, New York, NY 10027, USA; Australian Nuclear Science and Technology Organisation and University of Wollongong, Australia

**Keywords:** ultrafast X-ray diffraction, laser melting, pair distribution functions, inhomogeneous melting, liquid structure factors

## Abstract

X-ray structural science is undergoing a revolution driven by the emergence of X-ray Free-electron Laser (XFEL) facilities. The structures of crystalline solids can now be studied on the picosecond time scale relevant to phonons, atomic vibrations which travel at acoustic velocities. In the work presented here, X-ray diffuse scattering is employed to characterize the time dependence of the liquid phase emerging from femtosecond laser-induced melting of polycrystalline gold thin films using an XFEL. In a previous analysis of Bragg peak profiles, we showed the supersonic disappearance of the solid phase and presented a model of pumped hot electrons carrying energy from the gold surface to scatter at internal grain boundaries. This generates melt fronts propagating relatively slowly into the crystal grains. By conversion of diffuse scattering to a partial X-ray pair distribution function, we demonstrate that it has the characteristic shape obtained by Fourier transformation of the measured *F*(*Q*). The diffuse signal fraction increases with a characteristic rise-time of 13 ps, roughly independent of the incident pump fluence and consequent final liquid fraction. This suggests the role of further melt-front nucleation processes beyond grain boundaries.

## Abstract 2

Diffuse X-ray scattering is used to characterize the time dependence of the liquid phase emerging from femtosecond laser-induced melting of polycrystalline gold thin films using an XFEL. Its structure factor and partial pair distribution function confirm the liquid origin of the diffuse scattering. The liquid fraction increases with a characteristic rise-time of 13 ps.

## 1. Introduction

The classical physics of X-ray diffraction, the foundation of crystallography, assumes instantaneous scattering of X-ray waves, accelerating the core electrons within atoms, which radiate secondary waves that interfere to create a diffraction pattern in the far field. This interference produces measurable diffraction patterns encoding atomic positions in the scattering material. Under the Born–Oppenheimer approximation, each photon scattering event is instantaneous and captures a snapshot of atomic positions at a moment in time. When measuring many scattering events over a time period greater than the characteristic atomic vibration time in a material – such as during a synchrotron or laboratory source measurement – information is lost as distinct diffraction patterns are time averaged. This appears in practice as the Debye–Waller effect, where Bragg peak intensities are increasingly supressed at higher scattering angles. X-ray free-electron laser (XFEL) facilities produce intense, coherent ∼100 fs pulses of X-ray radiation that irradiate a sample for less than an atomic vibrational period and enable the phonons to be measured (Trigo *et al.*, 2013 ▸; Teitelbaum *et al.*, 2018 ▸). This is an essential component to pump–probe-type experiments in which a femtosecond laser pumps a sample while a synchronized X-ray probe measures the resulting structural changes after a fixed delay time with possible picosecond or better resolution. This has enabled a fundamentally new temporal regime for X-ray structural measurements. If the sample returns to an equivalent equilibrium state after each pump-and-probe cycle, it may be measured stroboscopically where multiple measurements are averaged for the same delay time to improve the signal-to-noise ratio. However, XFEL pulses are sufficiently bright that a parallel detector system can capture meaningful X-ray diffraction data in a single shot even if the sample is irreversibly altered or destroyed. Here we use this ‘diffraction before destruction’ concept (Neutze *et al.*, 2000 ▸; Chapman *et al.*, 2011 ▸) to investigate the melting of thin films of gold, a fundamental change of state from solid to liquid.

Melting and crystallization dynamics have been studied using molecular dynamics methods, for example, by Nose & Yonezawa (1986 ▸), Giret *et al.* (2013 ▸) and Molina & White (2022 ▸). Although the dynamics of the freezing can be complicated by the gradual assembly of unit cells of a crystal, melting releases latent heat and represents a sharp loss in structural order and increase of entropy. This makes melting the favourable direction across this phase boundary to study as there should be no entropic barrier to the formation of the liquid state. This work extends the data analysis from an experiment by Assefa *et al.* (2020 ▸) in which X-ray scattering was used to investigate the structural evolution of gold thin films undergoing melting by a femtosecond laser. Previous analysis of these data revealed spatial inhomogeneities in the melting process arising from the granular structure of the film. However, the analysis was limited to studying the temporal behaviour of the Bragg peaks representing crystalline (pre-melted) material. Here, this analysis is extended to study the diffuse (non-Bragg) scattering dynamics and applies both liquid structure factor and pair distribution function (PDF) methods to show that this increasing signal indeed arises from liquid gold.

## 2. Experimental procedure and theory

Laser-induced melting of a granular metal results from a sequence of events described by a ‘two-temperature’ model (TTM) of electrons and ions. The femtosecond pump laser interacts only with free electrons both within the electromagnetic ‘skin depth’ (a few nanometres) of the irradiated surface and close to the Fermi level in energy. Optical pulses of sufficient intensity will cause multiple excitations of this accessible subset of electrons and raise their temperature to thousands of degrees. This initial excitation is virtually invisible to an X-ray probe pulse which is sensitive mainly to the atomic core electrons. During a characteristic electron–phonon coupling time of ∼1 ps (Chen *et al.*, 2006 ▸; Wilson & Coh, 2020 ▸), these ‘hot electrons’ interact with core electrons and the energy introduced by the laser is transferred into heating the crystal lattice. Then other thermal transport mechanisms proceed. The TTM model of laser-induced metal heating has been previously verified in the work by Chen *et al.* (2006 ▸), Giret *et al.* (2013 ▸) and others. The main question of interest in this and other XFEL pump–probe studies of melting is *where does the electron-phonon coupling take place?* And hence where within the solid does the melting start? Free electrons move at the Fermi velocity around 10^6^ m s^−1^ so can travel several micrometres in the 1 ps coupling time, further than the size of the sample in many cases. In a perfect crystal at *T* = 0, electrons should travel as uninterrupted Bloch waves, but the rates of electron–lattice interactions will be expected to increase with the lattice temperature and the presence of defects, surfaces or interfaces in the metal, as discussed in the interpretation of transport measurements by Mayadas & Shatzkes (1970 ▸).

Assefa *et al.* (2020 ▸) reported femtosecond laser-induced melting of 300 nm-thick evaporated gold films significantly thicker than the optical penetration depth. These were melted using a 400 nm pump laser of variable intensity and measured using 100 fs probe X-ray pulses generated by the Pohang Accelerator Laboratory X-ray Free-Electron Laser (PAL-XFEL) with sufficient X-ray flux that the diffraction pattern could be measured in a single shot on a wide-angle area detector placed 50 mm behind the sample. More experimental details are given by Assefa *et al.* (2020 ▸). The gold samples were evaporated onto thin silicon nitride membrane windows in arrays that allowed automated positioning. Single-shot measurements were required because at high-fluence levels the laser melting of the film was found to shatter the windows, hence the diffraction pattern was safely recorded on the detector before any significant damage could take place. Pump–probe delay scans, with each delay point using a fresh window in an array, allowed Assefa *et al.* (2020 ▸) to track the conversion of the crystalline material of the thin films through the shape of its Bragg peaks. These peaks, especially the strong 111 first Bragg peak of polycrystalline gold, were found to split into two separate components, one remaining close to the starting position and one at a smaller momentum transfer (*Q*) for which the intensity built up over time. This lower diffraction angle peak was identified as gold heated to its melting point which underwent melting and released latent heat. The behaviour of the split peak clearly confirms an inhomogeneous melting process.

**Figure 1:**
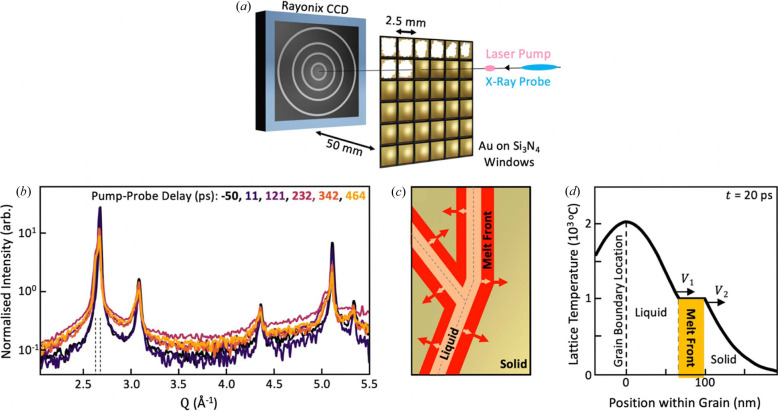
(*a*) An XFEL X-ray pulse is used to collect a diffraction pattern from a gold thin film after it has been pumped by an optical laser pulse. This pattern is collected in one shot on a Rayonix optically coupled charge-coupled device (CCD) 50 mm behind the sample. As the laser destroys the sample, each shot is taken using a unique sample window from a 2D array. (*b*) Diffraction intensity (log scale) from single pump–probe shots of monochromatic X-rays from PAL-XFEL with varying pump–probe delay times. The background has been measured and subtracted. Dashed lines indicate split Bragg peak positions after pumping. (*c*) Schematic of melting around grain boundary locations in a polycrystalline metal thin film, indicating the propagation of the laser-excited melt front as described by Assefa *et al.* (2020 ▸). (*d*) 1D spatial temperature distribution 20 ps after a spike of melt is generated at a grain boundary. The heat conducts into the grain with a melt-front velocity proportional to the heat flux, as proposed by Assefa *et al.* (2020 ▸). The temperature is fixed in the region where the metal is actively melting due to latent heat, resulting in a block of melting material sandwiched between two melt fronts moving at different velocities.

This inhomogeneous melting picture is consistent with other published studies of metal thin films prepared in different ways (Mo *et al.*, 2018 ▸; Siwick *et al.*, 2003 ▸; Hartley *et al.*, 2017 ▸; Milathianaki *et al.*, 2013 ▸). Assefa *et al.* (2020 ▸) proposed a two-step explanation involving electrons and the crystal lattice in the TTM described above. The innovation of the model was that the location of this electron–lattice coupling was spatially confined to the grain boundaries of the polycrystalline film. It has been known since the work by Mayadas & Shatzkes (1970 ▸) that the resistivity of metal thin films increases with the grain boundary density, which is the inverse of the grain size; this implies electron scattering is confined to these locations. Scattering at the grain boundaries implies that energy transfer is localized there too. Assefa *et al.* (2020 ▸) proposed that local heating of the grain boundaries initiates a melt front that propagates into the neighbouring grains, shown in Fig. 1 ▸(*c*). Because of the role of latent heat, there are two melt fronts, one for the material reaching the melting point and a second following where the latent heat release has completed the melting and the liquid phase starts. This traps a partially crystalline block of material at the melting point, which gives rise to the extra Bragg peak. A quantitative thermal diffusion calculation of this model after 20 ps is shown in Fig. 1 ▸(*d*), accounting for thermal conduction and latent heat following a sharp injection of heat at *t* = 0. The shifted low-*Q* Bragg peak observed in the experiment is attributed to the coloured band of material undergoing melting at the melting point temperature. As a function of increasing time delay, this peak was observed to become slightly narrower, indicating that the melt-front band was getting thicker. This is consistent with thermal transport calculations and allowed an estimate of the melt front velocity: *v*_melt_ ≃ 30 m s^−1^.

## 3. Appearance of liquid gold

**Figure 2:**
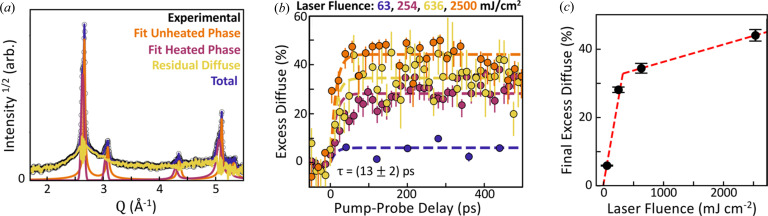
(*a*) Decomposition of the scattering intensity into Bragg and diffuse components. Two sets of closely spaced peaks model the diffraction of the crystalline phases while a broadband function models the diffuse component. (*b*) Integrated diffuse scattering intensity expressed as a fraction of the total measured scattering as a function of pump laser fluence and pump–probe delay. The signal is designated ‘excess’ because the crystal-diffuse part, measured at negative time delay, has been subtracted. Dashed lines are common fits to an exponential function to obtain the relaxation rate. (*c*) Fluence dependence of the asymptotic excess diffuse signal. Dashed lines indicate two fluence regimes separated by a critical fluence of 330 mJ cm^−2^.

The Bragg component of the scattering signal, discussed above, represents only crystalline phases, which are assumed to be non-melted. In addition to this, disorder and/or non-crystalline material generates diffuse scattering apparent as the broadband ‘background’ on which the Bragg peaks sit. In this report, we track the dynamics of the diffuse scattering in the experiment reported by Assefa *et al.* (2020 ▸) and show that, although a broadband in reciprocal space, this diffuse signal contains enough information to confirm its origin as liquid gold. To isolate the diffuse scattering, the split Bragg peaks were fit using Voigt functions and removed as shown in Fig. 2 ▸(*a*). These fits were unconstrained without consideration for the crystal lattice, instrument resolution or crystallite size to minimize any misfits of the Bragg peaks influencing the residual diffuse intensity. It should be emphasized that such binary classification of intensity as ‘Bragg’ and ‘diffuse’ will always contain some ambiguity near peak positions. Subtracting the average diffuse intensity for negative pump–probe delay times provides the ‘excess diffuse’ scattering generated as a result of the laser pulse excitation.

To track the dynamics of the diffuse scattering, the total excess diffuse intensity was integrated as a fraction of total scattering, designated the ‘excess diffuse fraction’ in Fig. 2 ▸(*b*), to give the fraction of the signal originating from the new non-crystalline phase assumed to be dominated by liquid gold. There is a clear rise in this diffuse signal at positive pump–probe delays, which has been fitted with the exponential time constant τ = 13 ± 2 ps common to all fluences. The asymptotic liquid fraction in Fig. 2 ▸(*c*) shows an increase as the pump laser fluence was increased from 63 to 2500 mJ cm^−2^, corresponding to energy doses from 108 to 4286 kJ kg^−1^ across the entire 300 nm sample. Although there are only four fluence values, the trend appears to start linearly but slows down after a critical fluence of 330 mJ cm^−2^ (566 kJ kg^−1^ dose), identified in Fig. 2 ▸(*c*). We are not able to clearly identify the acoustic oscillations in the diffuse signal that correspond to those observed in the Bragg peak intensities by Assefa *et al.* (2020 ▸), but this might be possible in future work. The linear regime is consistent with the melt-front ideas described above, which predict (in the first approximation) that the melt-front velocity should be proportional to fluence. Averaged over the whole film, the temperature rise expected from simple specific heat calculations by Assefa *et al.* (2020 ▸) is 2700 K for the critical fluence level, which would bring the sample to a boiling point of 3000 K. The sub-linear fluence dependence can therefore be attributed to partial ablation or vaporization of gold beyond the critical fluence level. It could also be attributed to hot electrons leaving the sample, as suggested by Molina & White (2022 ▸).

Although lacking long-range periodicity, liquid metals do exhibit short-range structure due to nearest-neighbour van der Waals type interactions (Chaikin & Lubensky, 2000 ▸). This can be observed using a PDF which contains well defined peaks at common inter-atomic distances within a material. For a liquid metal, this displays a strong nearest-neighbour peak at the minimum of the interatomic potential followed by a rapid decay of structural correlations over larger distances. The PDF, denoted *G*(*r*), where *r* is the interatomic distance, is generated from the diffraction pattern by first applying a linear transform to produce the reduced structure factor *F*(*Q*). This is followed by a sine Fourier transform to real space (Billinge, 2019 ▸):

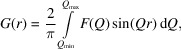

where the integration bounds represent the experimental measurement range and introduce ‘termination ripple’ artefacts to the PDF. As this equation is linear with respect to the input diffraction pattern, this can be applied to the diffuse scattering signal in isolation to ignore the crystalline phases if any diffraction contribution from the crystalline/non-crystalline interface region is considered negligible.

**Figure 3:**
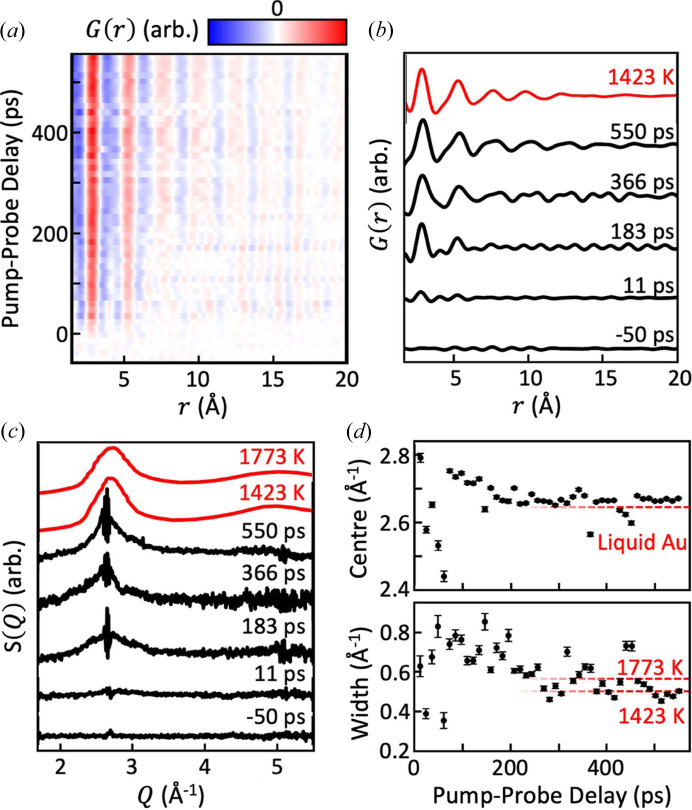
PDFs and excess diffuse scattering functions obtained at 636 mJ cm^−2^ pump laser fluence as a function of pump–probe delay time. (*a*) Waterfall plot where the peak broadening can be identified as a function of time delay. (*b*) Detailed plots at three representative time delays, together with a PDF calculated from the measured *F*(*Q*) at 1423 K (Waseda & Ohtani, 1974 ▸). (*c*) Excess diffuse scattering profile extracted by removal of the Bragg peak contributions and subtraction of the profile measured at negative time delay. The measured structure factor of liquid gold is shown at two temperatures in colour at the top of the panel. (*d*) Results of Gaussian fitting of the first maximum of the structure factor as a function of pump–probe delay time.

PDFs generated from the excess (over the negative time delay) diffuse scattering signals are shown as a function of pump–probe delay at 636 mJ cm^−2^ in Figs. 3 ▸(*a*) and 3 ▸(*b*). Owing to the very limited experimental *Q* range (1.5–5.5 Å^−1^) of the current XFEL measurements, these PDFs suffer from low resolution with broad features and strong termination ripples. A slight continuous evolution of the PDF with time delay, such as a slight broadening in time of the first maximum and a gradual disappearance of the fine structural features, can be observed, especially in Fig. 3 ▸(*a*). The quality of the PDFs is too poor to draw any quantitative conclusions, but the result is indicative of what might be seen in a future study with full resolution. Though it is possible that the observed broadening is due to imperfect Bragg–diffuse separation, it is conceivable that there is a signature of liquid gold with insufficient time to fully erase the information of its previously crystalline arrangement, with incompletely melted nanocrystalline regions of solid mixed with the liquid gold, for example. However, this is highly speculative due to the low resolution and strong termination artefacts of these data. The experimental PDFs are compared with the PDF calculated from the published *F*(*Q*), measured by Waseda & Ohtani (1974 ▸), under the same conditions (*i.e.* *Q* range and resolution) as the experimental data. Good qualitative agreement is observed between this classical liquid PDF and the experimental results.

The excess diffuse scattering signals are also shown directly as the reciprocal-space structure factor *F*(*Q*), following subtraction of the data at negative time delay, shown in Fig. 3 ▸(*c*). Despite some residue of the removed Bragg peaks, there is a clear resemblance with the liquid gold structure factor (Waseda & Ohtani, 1974 ▸) shown at the top of Fig. 3 ▸(*c*). The two temperatures, 1423 and 1773 K, show a slight difference especially in the width of the first maximum of the structure factor. As far as we know, this difference has not been discussed in the literature, but indicates the presence of slightly longer-range order at 1423 K, closer to the melting point of 1337 K.

We therefore fitted the laser-driven excess diffuse scattering signals to a simple Gaussian function to extract the peak centre and widths, which are presented in Fig. 3 ▸(*d*). The residue of the Bragg peaks was removed by excluding the 2.57 Å^−1^ < *Q* < 2.74 Å^−1^ range from the fits. No attempt has been made to exclude ‘bad shots’, a common feature of XFEL data where the beam steering during the X-ray lasing process fluctuates or is incorrect. Nevertheless, there is a clear trend lasting for the first 150 ps, where the peak width is larger and the centre is shifted to higher *Q*. The trends in the PDF mentioned above also lie in this temporal range. We note that this time scale is similar to the phonon transit time through the film, observed by Assefa *et al.* (2020 ▸) as oscillations with a period of 130 ± 10 ps. It is not surprising that the peak width is larger during the early time period, when the temperature inhomogeneities are more pronounced, because the X-ray beam is averaged over the entire film thickness. After this equilibration period, both the peak width and the centre reach values close to those of the liquid, measured by Waseda & Ohtani (1974 ▸), with a possible acoustic oscillation visible in the width. This supports our interpretation of the majority of the diffuse signal coming from liquid gold.

## 4. Implications for the melt-front picture of inhomogeneous melting

In the work by Assefa *et al.* (2020 ▸), the Bragg peak profiles in these experimental data were used to propose the melt-front model of the inhomogeneous metal melting described above. The proposed model of melt-fronts propagating relatively slowly (∼30 m s^−1^) into the grains is consistent with the initial linear increase of the excess diffuse scattering (originating from the melted gold) seen in this analysis. Similarly, the initial linear fluence dependence is expected from the melt-front model. The time constant of the liquid fraction increase, τ = 13 ± 2 ps (roughly independent of laser pump fluence), can be taken as an estimate of the melting time. This corresponds roughly with the onset time of the intermediate diffraction peak of Assefa *et al.* (2020 ▸). A roughly linear rise and similar rise-time value were observed in recent XFEL experiments on melting Pd thin films by Suzana *et al.* (2023 ▸). Previous measurements of laser-induced Au thin film melting have reported melt times of 7.3 ps for 20 nm films (Siwick *et al.*, 2003 ▸) and 20 ps for 30 nm films (Mo *et al.*, 2018 ▸). These can all be understood within the melt-front model of inhomogeneous melting starting at defects.

We note that, from the previous estimate of the velocity, the melt-front travels a distance of less than 1 nm within the rise time, which is a small fraction of the estimated 160 nm grain size in these thin films. A possible explanation for this could be that there are other melt-front nucleation sites in addition to the grain boundaries. Any electron scattering processes, leading to electron phonon coupling, could initiate melting, for example. The scaling of the rate of formation of liquid with laser fluence is consistent with the expected increase of melt-front velocity with fluence, modified by the onset of ablation discussed above. Following this relaxation time, the amount of liquid stays constant over time, but still increases with the pump fluence.

In summary, we extracted the diffuse scattering signal from gold thin films undergoing laser-induced melting, extracted the structure factor and generated the corresponding PDFs. These are consistent with diffuse scattering originating mostly from liquid gold. There appears to be a slight evolution to the PDFs with delay time, but the small range of scattering data limits the real space resolution enough to preclude a more detailed interpretation. However, this hints at the level of possible information on melting dynamics that could be extracted from dedicated XFEL experiments, once fully optimized for PDF. Larger experimental measurement ranges are already available and will likely increase further with the evolution of XFEL facilities. Our results support the picture of an inhomogeneous spatial distribution of nucleation of melt fronts found in the previous analysis of diffraction peaks by Assefa *et al.* (2020 ▸), with the diffuse signal increasing linearly over time and then saturating. The time taken for the diffuse signal to saturate (τ = 13 ± 2 ps) is independent of the total melting fractions, suggesting the role of other melt front nucleation sites beyond grain boundaries. It takes slightly longer for the liquid structure factor to reach the equilibrium width and position expected from liquid gold.
